# NAD^+^ Metabolism and Diseases with Motor Dysfunction

**DOI:** 10.3390/genes12111776

**Published:** 2021-11-09

**Authors:** Samuel Lundt, Shinghua Ding

**Affiliations:** 1 Dalton Cardiovascular Research Center, University of Missouri-Columbia, Columbia, MO 65211, USA; slhvr@mail.missouri.edu; 2 Interdisciplinary Neuroscience Program, University of Missouri-Columbia, Columbia, MO 65211, USA; 3 Department of Biomedical, Biological and Chemical Engineering, University of Missouri-Columbia, Columbia, MO 65211, USA

**Keywords:** NAD^+^, Nampt, energy metabolism, motor neuron diseases, motor dysfunction

## Abstract

Neurodegenerative diseases result in the progressive deterioration of the nervous system, with motor and cognitive impairments being the two most observable problems. Motor dysfunction could be caused by motor neuron diseases (MNDs) characterized by the loss of motor neurons, such as amyotrophic lateral sclerosis and Charcot–Marie–Tooth disease, or other neurodegenerative diseases with the destruction of brain areas that affect movement, such as Parkinson’s disease and Huntington’s disease. Nicotinamide adenine dinucleotide (NAD^+^) is one of the most abundant metabolites in the human body and is involved with numerous cellular processes, including energy metabolism, circadian clock, and DNA repair. NAD^+^ can be reversibly oxidized-reduced or directly consumed by NAD^+^-dependent proteins. NAD^+^ is synthesized in cells via three different paths: the de novo, Preiss–Handler, or NAD^+^ salvage pathways, with the salvage pathway being the primary producer of NAD^+^ in mammalian cells. NAD^+^ metabolism is being investigated for a role in the development of neurodegenerative diseases. In this review, we discuss cellular NAD^+^ homeostasis, looking at NAD^+^ biosynthesis and consumption, with a focus on the NAD^+^ salvage pathway. Then, we examine the research, including human clinical trials, focused on the involvement of NAD^+^ in MNDs and other neurodegenerative diseases with motor dysfunction.

## 1. Introduction

Neurodegenerative diseases are disorders of the nervous system characterized by the progressive destruction of neurons. The symptoms of a neurodegenerative disease depend on where the degeneration is occurring, with cognitive and motor deficits being especially noticeable [1,2]. Motor neuron diseases (MNDs) are a type of neurodegenerative disease that involves the degeneration of motor neurons from the brain, brain stem, or spinal cord, and results in progressive motor dysfunction [3]. Motor neurons can be classified as upper motor neurons, which originate in the motor cortex, and lower motor neurons, which originate in the brainstem or spinal cord and innervate skeletal muscles [4]. The most common MND is amyotrophic lateral sclerosis (ALS). Charcot–Marie–Tooth disease and spinal muscular atrophy (SMA) are also common MNDs, though less prominent than ALS. Other neurodegenerative diseases that also cause motor problems can occur without motor neuron degeneration. These neurodegenerative movement disorders have neurodegeneration of certain brain regions, which results in overt motor symptoms. These disorders include Parkinson’s disease and Huntington’s disease [5].

**Figure 1:**
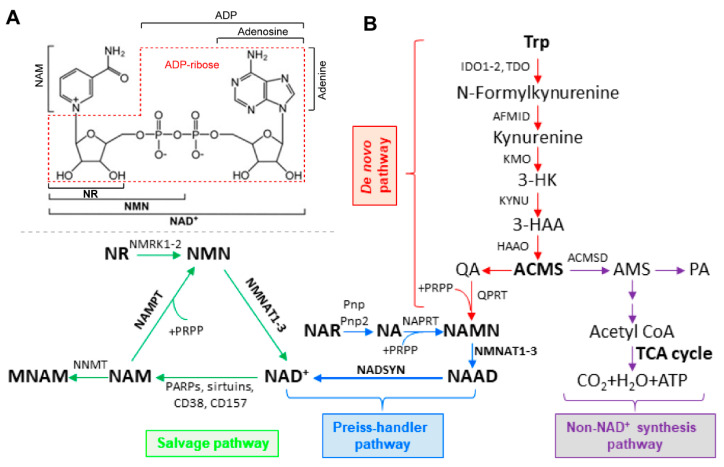
NAD^+^ structure (**A**) and biosynthetic pathways in mammalian cells (**B**). 3-HK: 3-Hydroxyl-Kynurenine; 3-HAA: 3-Hydroxyl-Anthranillic Acid; AMS: α-aminomuconate semialdehyde; MNAM: methyl-nicotinamide; NAR: Nicotinic Acid Riboside; NNMT: Nicotinamide N-methyltransferase; PA: Picolinic Acid; Pnp: Purine Nucleoside Phosphorylase; PRPP: Phosphoribosyl pyrophosphate.

Nicotinamide adenine dinucleotide (NAD^+^) is one of the most abundant metabolites in the human body and is predominantly synthesized through the NAD^+^ salvage pathway in mammalian cells, where nicotinamide phosphoribosyltransferase (Nampt) is the rate-limiting enzyme (Figure 1). NAD^+^ is a critical cofactor in numerous reactions, with its role in energy metabolism (glycolysis, TCA cycle, oxidative phosphorylation, and fatty acid oxidation) being the most well-known. NAD^+^ also functions as a substrate for certain NAD^+^-consuming enzymes, including sirtuins, poly-(ADP-ribose) polymerases (Parps), CD38/157, and sterile α and TIR motif-containing protein 1 (SARM1). This direct consumption of NAD^+^ is utilized for many different activities, such as DNA repair, the circadian clock, and protein deacetylation [6]. Thus, the maintenance of intracellular NAD^+^ levels is crucial for cellular functions and survival.

There is growing evidence that NAD^+^ can have an impact on neurodegeneration [6,7,8]. NAD^+^ and NAD^+^ biosynthetic enzymes are essential for neuronal health and survival, and this has led to NAD^+^ being investigated for a possible role in different neurodegenerative diseases, including Alzheimer’s disease and Parkinson’s disease, with findings suggesting NAD^+^- or NAD^+^-synthesizing enzymes could be involved in pathological development or therapeutic strategies [6,7]. NAD^+^-consuming enzymes, such as sirtuins, Parps, and SARM1, are also being investigated for their role in neurodegenerative diseases.

In this review, we will first review intracellular NAD^+^ homeostasis, focusing on the NAD^+^ salvage pathway, and consumption. Then, we will examine the involvement of NAD^+^ in different MNDs, including ALS and Charcot–Marie–Tooth disease, and discuss how NAD^+^ biosynthesis and availability may be involved in the development and progression of the MND as well as any therapeutic potential NAD^+^ may have. We will also discuss the role of NAD^+^ in other neurodegenerative movement disorders with strong genetic components and significant motor impairment, including Parkinson’s disease and Huntington’s disease.

## 2. Cellular NAD^+^ Homeostasis

Structurally, NAD^+^ is a molecule of nicotinamide (NAM) attached to a molecule of adenosine diphosphate ribose (ADP-ribose) (Figure 1A). Given that NAD^+^ is involved in many cellular activities, intracellular NAD^+^ levels are important for cells to function normally. While NAD^+^ levels appear to be relatively stable in cells, NAD^+^ homeostasis is a very dynamic process. Maintaining NAD^+^ homeostasis requires a balance between NAD^+^ biosynthesis and NAD^+^ consumption. In energy metabolism, NAD^+^ can be reversibly oxidized or reduced. This usage does not contribute to fluctuations in NAD^+^ levels. NAD^+^ levels can be affected by NAD^+^-consuming enzymes, and the by-products of the reactions are free NAM and ADP-ribose [9]. In healthy states, NAD^+^ levels are well maintained while in pathological conditions, NAD^+^ homeostasis can become disrupted, causing many problems including metabolic dysfunction and oxidative stress [9,10,11]. Therefore, NAD^+^ homeostasis is determined by the balance between biosynthesis and consumption.

### 2.1. Intracellular NAD^+^ Biosynthesis

In mammalian cells, NAD^+^ can be synthesized using different metabolites and precursors (Figure 1A). Vitamin B3, also called niacin, is the name given to the starting metabolite in NAD^+^ biosynthesis and can refer to two different vitamers: nicotinamide (NAM) and nicotinic acid (NA) [12]. These metabolites can be utilized in three different pathways for NAD^+^ synthesis: the de novo, Preiss–Handler, and salvage pathways, with the NAD^+^ salvage pathway being responsible for the majority of NAD^+^ production in mammalian cells (Figure 1B) [13]. The NAD^+^ salvage pathway consists of only two steps and begins with the metabolite NAM. In the first step, NAM is converted into nicotinamide mononucleotide (NMN) by Nampt, the rate-limiting step [14]. In the CNS, Nampt expression appears to be mostly restricted to neurons [15]. In the second step, NMN is converted to NAD^+^ by nicotinamide mononucleotide adenylyltransferase (Nmnat) [16]. There are three Nmnat isoforms (Nmnat1–3) and each have different sub-cellular localizations, with Nmnat1 being found in the nucleus, Nmnat2 in the cytosol and Golgi, and Nmnat3 in the mitochondria and cytosol [7,17]. Nmnat2 is highly expressed in the CNS and is very important to axon survival and synaptic function [6,18,19,20]. NMN can also be formed in an Nampt-independent manner, using the metabolite nicotinamide riboside (NR), by nicotinamide riboside kinases (Nrk1-2), though Nrk expression is more tissue specific compared to the more ubiquitous expression of Nampt [21,22].

Total intracellular NAD^+^ levels can be measured using available assay kits, but recently, new techniques have allowed for measuring NAD^+^ in different sub-cellular compartments in live cells [23,24]. The sub-cellular compartments have distinct NAD^+^ pools that remain relatively stable, with the cytosol and nucleus having similar NAD^+^ levels but the mitochondria having much higher NAD^+^ levels [24]. Considering the different sub-cellular localizations of the Nmnat isoforms (nucleus, cytosol, and mitochondria), the existence of different NAD^+^ pools in these areas is unsurprising. Whether both of the enzymes of the NAD^+^ salvage pathway, Nmnat and Nampt, are expressed in each of these sub-cellular regions has been less understood.

There is a general consensus that the synthesis of NAD^+^ via the salvage pathway, from NAM to NAD^+^, can occur in the nucleus and cytosol but disagreement concerning the mitochondria [6,7,9,25,26]. Questions remain regarding whether Nampt is also present in the same sub-cellular compartments as Nmnat1–3. Nampt was found to be present in the nucleus and cytosol, with proteins levels varying depending on the cell cycle [27]. There has been some conflicting evidence over Nampt in the mitochondria. Initially, Nampt was detected in mitochondria fractionates [28]. However, following studies did not detect Nampt in mitochondrial fractionates and also found that mitochondrial NAD^+^ was not depleted following exposure to FK866, an Nampt inhibitor [29]. A later study did find that FK866 treatment results in mitochondrial NAD^+^ depletion [24]. This difference may be due to the different cell line models used.

The presence of Nampt in neuronal mitochondria is more established. Nampt was present in mitochondria isolated from neuron-glia co-cultures, with expression controlled by the protein kinase Cε-AMP-activated protein kinase (APMK) pathway. Immunostaining showed Nampt co-localizes with CoxIV, a mitochondrial marker, in the cortex of adult rat brains [30]. Recently, Nampt was observed in the mitochondrial fractionates of cortical neurons in mice. Furthermore, Nampt and Nmnat3 were found to localize in the same mitochondrial region, the mitochondrial matrix, demonstrating there is an intrinsic machinery for NAD^+^ synthesis in neuronal mitochondria [31]. Additionally, the overexpression of Nampt in the nucleus/cytosol or the mitochondria of primary cortical neurons produced similar rates of survival following oxygen-glucose depravation [31]. Overall, Nampt is expressed in the mitochondria of neurons and this mitochondrial Nampt can improve neuronal survival.

A second pathway for NAD^+^ synthesis is the Preiss–Handler pathway. The Preiss–Handler pathway has three steps. In the first step, NA is converted to nicotinic acid mononucleotide (NaMN) by nicotinic acid phosphoribosyltransferase (Naprt) [13]. NaMN is converted into nicotinic acid adenine dinucleotide (NaAD) by Nmnat. Nmnat1-3 can recognize both NMN and NaMN and use them as substrates. NaAD is then formed into NAD^+^ by NAD synthetase (Nadsyn) [16]. The Preiss–Handler pathway is predominantly expressed in the liver, kidney, and intestines and has not been found to be highly expressed in the CNS [32,33].

The de novo pathway starts with the amino acid tryptophan and is converted into quinolinic acid (QA) after five enzymatic steps, with the initial step being performed by either tryptophan-2,3-dioxygenase (Tdo) or indoleamine-2,3-dioxygenase (Ido1-2) [13]. The next steps are controlled by Arylformamidase (Afmid), kynurenine 3-monooxygenase (Kmo), kynureninase (Kynu), and 3-hydroxyanthranilate 3,4 dioxygenase (Haao), leading to the production of QA from the spontaneous degeneration of α-amino-β-carboxymuconate-ε-semialdehyde (ACMS) [6,16]. QA is then converted into NaMN via quinolinate phosphoribosyltransferase (Qprt) [13]. After NaMN is formed, it can enter the Preiss–Handler pathway to form NAD^+^ [7]. The enzymes in the de novo pathway are expressed in the CNS but differ between neural and glial cells. Microglia express all the necessary enzymes for de novo NAD^+^ synthesis but astrocytes have not been found to express high levels of de novo pathway enzymes though, under certain circumstances, can produce large amounts of QA. Neurons, despite expressing Ido1, do not express the needed enzymes for the complete production of NAD^+^ from tryptophan. This may be due to many of the intermediate metabolites being potentially toxic to neurons [34].

### 2.2. Nampt Expression in the CNS

Considering the majority of NAD^+^ is produced using the NAD^+^ salvage pathway in mammalian cells and that Nampt is the rate-limiting enzyme, understanding Nampt expression throughout the CNS is important. Research has found mammals have two different forms of Nampt, intracellular Nampt (iNampt) and extracellular Nampt (eNampt), and they can be discriminated from each other by different molecular weights [35,36]. The distribution of iNampt in mice differs between tissue types. Initially, iNampt expression was undetected in the brain, with high expression levels in brown adipose tissue, liver, and kidney; intermediate level in the heart; and low levels in white adipose tissue, lung, spleen, testis, and muscle [35]. However, later research determined that iNampt was present in the mouse brain. iNampt protein is primarily expressed in neurons in the hippocampus and cortex but is not detectable in glial cells in the mouse brain, and, additionally, some endothelial cells also express iNampt [15,37]. Further research showed that iNampt was expressed in cultured astrocytes though at a considerably lower level compared to the high level in neurons [37]. In the hippocampus, neurons in the granule layer, but not along the sub-granular zone of the dentate gyrus, express Nampt, and it has been suggested that a large number of neural stem/progenitor cells have the highest expression of Nampt in the hippocampus [38]. The cerebellum also has expression of iNampt, specifically in Purkinje cells, granule cells, and cells in the molecular layer [39].

eNampt, which is post-translationally modified from iNampt, has been found in many tissues throughout the body, including in the brain and in the blood [35,36]. Many studies have shown that eNampt can be non-classically secreted by many different cell types, such as adipose tissue, hepatocytes, leucocytes, and macrophages [35,40,41,42,43]. It was reported that eNampt is more enzymatically active than iNampt [35]. Following ischemia, neurons can secrete eNampt in the CNS and eNampt was selectively induced after ischemia in both the fiber bundles of the striatum and the corpus callosum [36]. Similar to NAD^+^ levels, eNampt levels decline with age. Extracellular vesicles that contain eNampt can be taken up by neurons and increase intracellular NAD^+^ and NMN levels. It has also been shown that treating aged mice with extracellular vesicles containing eNampt significantly extended lifespan and improved motor behavior [44]. In ALS patient spinal cords, eNampt expression was elevated [45]. However, further investigation is needed to determine how eNampt may be involved in ALS and MNDs.

### 2.3. Cellular Consumption of NAD^+^

NAD^+^ is well-known for the important role it serves in metabolic oxidation and reduction reactions but also is an essential cofactor for many other reactions within mammalian cells. NAD^+^ is cleaved in these reactions, with NAM and ADP ribose/cyclic ADP ribose being formed as a result. The primary NAD^+^-consuming enzymes are the sirtuins (Sirts), Parps, CD38/CD157, and SARM1 [46]. Cellular NAD^+^ levels are significantly impacted by reducing or increasing Nampt expression [45,47,48,49,50]. Both NAD^+^ and Nampt levels appear to decline naturally during aging [51]. Changes in NAD^+^ availability can also significantly impact the activity and expression of these NAD^+^-dependent enzymes.

There are seven Sirt proteins (Sirt1-7), located sub-cellularly in the nucleus (Sirt1, 6, 7), cytosol (Sirt2), and mitochondria (Sirt3, 4, 5) [6]. Sirts are NAD^+^-dependent deacetylases and are involved in transcription regulation, mitochondrial metabolism, autophagy, and apoptosis [9]. Sirt1 is the most widely investigated Sirt and is involved in the circadian cycle of Nampt expression [25]. Sirt1 activation exhibits neuroprotective effects when NAD^+^ is available [52]. Sirt1 may also be a protective factor in neurodegenerative diseases, such as Alzheimer’s disease, Parkinson’s disease, and ALS [53,54,55,56]. Overexpression of Sirt1 improved neuronal growth and survival, even in the presence of β-amyloid, a toxic peptide primarily associated with Alzheimer’s disease [54]. Reducing Nampt expression impacts Sirt expression and activity. Sirt1 and Sirt6 expression levels were increased and Sirt3 decreased, following Nampt deletion from skeletal muscle [47]. Global deletion of Nampt elevated Sirt1 expression and reduced Sirt3, Sirt4, and Sirt5 expression in mouse livers and higher levels of acetylation were detected [57]. When Nampt was deleted from projection neurons, no changes in Sirt1 or Sirt3 levels were detected but increased acetylation was found, indicating a possible decrease in Sirt activity [45]. Increasing Sirt1 activity, either through NAD^+^ precursor treatment or inhibiting other NAD^+^-consuming enzymes, extended the lifespan of mice [58]. The primary benefits of Sirt activity may come from Sirt6. Global overexpression of Sirt6, but not Sirt1, lengthened lifespan and improved motor behavior. Sirt6 overexpression also enhanced the activity of metabolic pathways, such as gluconeogenesis, in aged mice [59].

Parps are another major consumer of cellular NAD^+^. The Parp family of proteins are involved with DNA repair, genome maintenance, and cell death [16,23]. Only four Parps (Parp1, 2, 5a, and 5b) transfer multiple ADP-riboses; the rest only transfer one and can also be referred to as mono-ADP-ribosyltransferases (Marts) [6,9]. Parp1 activity is estimated to make up over 80% of cellular Parp activity [16,60]. In contrast to Sirts, most research indicates that Parp inhibition can prove beneficial. Overactivation of Parp1 can lead to a specific form of cell death, called parthanatos, which is characterized by excess addition of poly-ADP-ribose (PAR) polymers (PARylation) and can quickly deplete cellular NAD^+^ [6,23,60,61]. Inhibition of Nampt reduced resistance to Parp1-induced cell death while overexpression enhanced resistance [62]. Elevating NAD^+^ levels through treatment with intermediates was also able to improve neuron survival against Parp1 overactivation and depleting NAD^+^ in neurons produced responses similar to Parp1 activation [63]. Parp1 expression is lowered in individuals after losing weight [64]. Curiously, PARylation increase but NAD^+^ levels decrease with age, indicating that Parp activity may contribute to the NAD^+^ decline that occurs during aging [58]. Parp1 overactivation may cause the lower NAD^+^ levels observed in mdx mice, a model for muscular dystrophy, but NR treatment was able to reduce Parp1 activity [65]. Elevated Nampt levels may inappropriately activate Parp1. Increased Nampt expression and PARylation occur following stroke or activation of the immune system [66,67]. Deletion of Nampt from projection neurons had no effect on PARylation, possibly because NAD^+^ levels were reduced [45]. If Parp1 is not overactive, NAD^+^ supplementation can actually improve the DNA repair controlled by Parps [68].

CD38 and CD157 are extracellular membrane-bound glycohydrolases and are important in regulation of Ca^2+^ second messenger signaling [9,25]. CD38 is a major consumer of NAD^+^ in the brain, with NAD^+^ levels increased 10 times in CD38 knockout mice [69]. Surprisingly, loss of CD38 activity does not prevent axonal degeneration even though NAD^+^ levels are elevated [70]. There is some evidence suggesting that the age-related decline in NAD^+^ levels could be the direct result of upregulated CD38 expression with age. In mice, multiple tissue types showed an increase in CD38 expression with increasing age whereas other NAD^+^ consumers did not change, or even decreased [71]. The effect of CD38 on different types of brain injury has also been investigated. Following a traumatic brain injury, CD38 knockout mice had slower recovery of motor and behavioral functions than wild-type mice. Loss of CD38 also reduced the microglial activation in the lesion area that can be neuroprotective, especially in the days immediately following the injury [72]. The impact of CD38 on stroke is less inconclusive. There is evidence to suggest CD38 plays an important role in neuronal recovery after an ischemic event [73]. However, CD38 knockout mice were able to resist NAD^+^ depletion and have Nampt expression return to basal levels more quickly following an ischemic event [74]. More research is needed to clarify the effect CD38 has following brain injury, specifically if CD38 activity is beneficial or harmful.

SARM1 is an NAD^+^ hydrolase in the TIR-domain family, with the TIR domain directly binding NAD^+^ [75,76,77]. SARM1 executes and is required for axonal degeneration following injury, also referred to as Wallerian degeneration, and causes an NAD^+^ depletion. Increased Nmnat expression can prevent both SARM1 activation and NAD^+^ depletion, indicating that the interaction between Nmnat and SARM1 is important for axonal survival [78]. Nmnat2 deficiency leads to widespread axonal loss, muscle denervation, and motor impairments, but when SARM1 is also deleted, these deficits are eliminated, and the mice appear to be comparable to wild-type mice [79]. Interestingly, activation of SARM1 may not require a physical injury but rather a change to Nmnat2 availability. Impairing the transport of Nmnat2 from the nucleus to the axon was enough to induce SARM1-dependent axon degeneration, possibly by altering the NMN/NAD^+^ ratio [80]. Recently, it was proposed that SARM1 detects Nmnat activity. This is supported by the finding that SARM1 can interact with NMN and NAD^+^, with NAD^+^ serving as an inhibitor and NMN as an activator [81]. The idea that SARM1 is a detector of Nmnat activity is further demonstrated by the finding that NaMN, which can be converted to NaAD by Nmnat in the Preiss–Handler pathway, also has an inhibitory effect on SARM1 [82]. This had led to mutations affecting SARM1 activity being investigated for a role in neurodegenerative diseases. Constitutively active SARM1 mutants, which can be over 10 times more active than wild-type SARM1 and not influenced by NMN levels, have been found in human patients with motor nerve diseases. The mutations alone are capable of inducing neurodegeneration and motor dysfunction [83,84]. The mechanisms involved in SARM1-dependent axon degeneration have recently been reviewed and suggest that intracellular Ca^2+^ influx and energy depletion are likely the downstream effects of SARM1 activation [85]. These recent findings indicate that research into SARM1 and the role it plays in various neurodegenerative diseases could provide novel insights into disease progression and development.

## 3. NAD^+^ and Diseases with Motor Dysfunction

### 3.1. Amyotrophic Lateral Sclerosis

ALS is the most common adult-onset MND, and is a progressive neurological disorder, characterized by the degeneration of motor neurons, limb weakness, paralysis, and, eventually, death [6]. ALS cases are separated into sporadic and familial. Familial ALS accounts for 10% of cases with the other 90% considered sporadic. There is no difference in the disease phenotype between familial and sporadic ALS. The direct cause of ALS has not been determined yet, though mitochondrial dysfunction, oxidative stress, and neuroinflammation, along with other impairments, have been observed in ALS and are being investigated [86,87]. Familial cases are caused by mutations to specific genes, including SOD1, TARDBP, FUS, and C9orf72. Of these, SOD1 mutations have been the most widely investigated. The majority of ALS research using animal models has focused on familial ALS, with SOD1^G93A^ mouse models being the most commonly used [88].

A possible role for NAD^+^ in ALS was first indicated from alterations to NAD^+^-related metabolites in the de novo pathway, with tryptophan, kynurenine, and QA levels being higher in the serum and CSF of ALS patients [89]. However, as the vast majority of NAD^+^ in cells is produced from the salvage pathway, this may indicate changes to overall NAD^+^ homeostasis in ALS. In fact, human ALS patients do have indications of NAD^+^ homeostatic disruption. ALS patients have reduced serum and CSF NAM levels [90]. Spinal cords of wobbler mice, a sporadic ALS mouse model, had decreased NAD^+^ levels [91]. NAD^+^ levels were significantly lower in the spinal cord, but not the brain, at symptom onset in SOD1^G93A^ mice and were significantly lower in both the spinal cord and the brain in the later stages of the disease [92]. Overall, this indicates that across animal models and human patients, normal NAD^+^ availability is impaired in ALS and reduced NAD^+^ levels may be a progressive aspect of ALS disease development.

There is also evidence that the NAD^+^ salvage pathway is altered in ALS. In motor neurons, loss of Nampt produced widespread dysfunction. The deletion of Nampt from projection neurons produced motor impairments similar to ALS, with eventual hindlimb paralysis and death. It also caused neuromuscular junction (NMJ) functional deficits and the destruction of NMJs [45,93]. Spinal cords of Wobbler mice had significantly reduced Nmnat2 expression prior to symptom onset and during disease progression [91]. Increasing activity of NAD^+^ salvage pathway enzymes, specifically Nampt, is beneficial to ALS motor neurons. SOD1^G93A^ ALS mice treated with P7C3, a proposed Nampt activator, had improved motor neuron survival and walking gait [94]. These results indicate that NAD^+^ salvage pathway enzymes are critical for motor neurons and increasing Nampt activity can enhance motor neuron survival in ALS.

There is direct evidence that the expression of NAD^+^ salvage pathway enzymes is altered in human ALS patients. In spinal cord samples, mRNA levels for Nmnat2 and Nampt are significantly different compared to control samples. Nmnat2 mRNA was significantly decreased while Nampt mRNA was significantly increased. The expression of other NAD^+^ biosynthetic enzymes was not altered. Consistent with the mRNA results, Nmnat2 protein expression in ALS spinal cords was also significantly lower [95]. There was also an increase in total Nampt protein levels, though iNampt and eNampt were altered differently. In ALS spinal cords, iNampt expression was reduced while eNampt expression was elevated compared to controls, resulting in a significantly different eNampt/iNampt ratio [45]. This indicates that decreased iNampt in motor neurons may be impairing normal NAD^+^ biosynthesis and result in enhanced eNampt secretion from other tissues and/or uptake by motor neurons to correct for this deficit. More than other NAD^+^ biosynthetic enzymes, Nampt and Nmnat2 are especially important to neurons and having both affected in ALS patient spinal cords indicates that the activity of the NAD^+^ salvage pathway may be an important target for treating ALS. Future clinical investigations for ALS should focus on how the NAD^+^ salvage pathway, especially Nampt, may be involved in either the development or progression of ALS.

With NAD^+^ levels and the NAD^+^ salvage pathway being impaired in ALS, increasing the availability of NAD^+^ is important. The easiest way to raise NAD^+^ levels is through the direct administration of NAD^+^ or an NAD^+^ precursor metabolite, usually NAM, NMN, or NR. In vitro, treating Wobbler motor neurons with NAD^+^ was able to increase neurite length [96]. The SOD1^G93A^ mice gut microbiome exhibited impaired NAM metabolism. Increasing NAM levels, either by augmenting the gut microbiota or with direct NAM administration, was able to enhance motor performance and survival [90]. Familial and sporadic ALS motor neurons developed from induced pluripotent stem cells (iPSCs) had improved mitochondrial function and neuron morphology following NAM treatment [97]. NR or NMN administration ameliorated motor function, motor neuron health, and lengthened the lifespan of ALS mice and following Nampt deletion from motor neurons [45,93,95,98]. Dietary supplementation using NAD^+^ precursors may also positively affect gene expression. NR or NMN supplementation enhanced expression for antioxidant defense and metabolic flexibility and reduced expression of denervation markers [95,99]. ALS mice placed on an NR diet had a significant increase in brain Nampt and Nmnat3 expression compared to ALS mice not provided NR [98]. This indicates that elevating NAD^+^ with an NAD^+^ precursor may be a valuable therapeutic approach for many ALS types.

While ALS is characterized by the degeneration and loss of motor neurons, glial cells, especially astrocytes, are also being investigated for a role in the development and progression of ALS. Overexpression of Nampt in astrocytes, either cytosolically or mitochondrially, was able to negate the toxic effects that astrocytes can have on motor neurons in ALS. This could be due to improving the response to oxidative stress by reducing reactive oxygen species [100]. Similarly, NMN or NR treatments were able to counter the toxic effects that ALS astrocytes can have on motor neurons [99]. Interestingly, the expression of NAD^+^ salvage pathway proteins in spinal cord astrocyte cultures from SOD1^G93A^ ALS mice were not significantly different from control astrocytes [100]. Though, this could be due to astrocytes relying more on glycolysis for energy and not oxidative phosphorylation, which requires more NAD^+^. NR treatment decreased activation of microglia and astrocytes in spinal cords of SOD1^G93A^ mice, suggesting that administering NAD^+^ precursors may also reduce the neuroinflammation observed in ALS [92,95].

The benefits of enhanced NAD^+^ availability may be due to increased activity of NAD^+^-consuming proteins, with Sirt activity likely being very important. Administration of resveratrol, a molecule that can activate Sirt1, or CNS Sirt1 overexpression can improve motor nerve function, slow disease progression, and lengthen the lifespan of ALS transgenic mice [56,101,102]. However, ALS mice treated only with pterostilbene, an Sirt1 activator, were not protected from motor dysfunction and did not have longer survival [92]. The protective effect of NAD^+^ could be through Sirt3 or Sirt6. The overexpression of Sirt3 or Sirt6, but not Sirt1, can improve motor neuron survival [99,100]. Knockdown of Sirt3 can produce ALS-like effects in culture and an Sirt3 activator can reverse the metabolic defects observed in ALS motor neurons [97]. Sirt6 may be the most important Sirt with respect to ALS, because the protective effects of NAD^+^ precursors were eliminated with the knockdown of Sirt6 expression [99]. Additionally, Sirt6, but not Sirt3, expression was significantly reduced in the spinal cords of human ALS patients [95]. Nevertheless, there are differences in Sirt expression between ALS mice and human patients. Sirt3 expression was reduced in SOD1^G93A^ spinal cords but elevated in ALS human spinal cords [103]. These differences between humans and animal models need to be better understood so that potential therapies identified using animal models can be effectively utilized by human patients. Parp activity could also be responsible for the changes to NAD^+^ levels observed in ALS. Parp-specific inhibitors have shown positive results in preventing the toxic accumulation of TDP-43 [104]. Elevated nuclear PAR was detected in the spinal cords of ALS patients, indicating possible increased Parp1 or Parp2 activity. Treating ALS primary spinal cord neurons with Veliparib, a Parp1/2 inhibitor, prevented TDP-43 toxicity [105]. Together, these findings indicate that Sirts and Parps are involved in ALS and that any treatments that augment NAD^+^ availability should also investigate how elevating Sirt activity or reducing Parp activity may lead to further benefits.

More recently, a role for SARM1 in ALS development and progression has been investigated. While the importance of the interaction between SARM1 and Nmnat2 on axonal health has been established, there have been some conflicting findings regarding SARM1 and ALS [85]. Inhibiting SARM1 in TDP-43 ALS models prevented motor neuron degeneration and axon loss, and improved innervation of hindlimb skeletal muscle [106,107]. However, in SOD1^G93A^ mice, SARM1 knockout did not enhance motor nerve function or prevent motor neuron degeneration [108]. While SARM1 may play a role in motor neuron survival in some ALS types, there does not appear to be any impact on motor function. Motor function was not ameliorated in either TDP-43 or SOD1^G93A^ mice if SARM1 was deleted [107,108]. Recently, a possible role for SARM1 in the development of ALS has been found. SARM1 mutations may cause an elevated risk for developing ALS while also exacerbating the disease development. Unique gain-of-function mutations are more prevalent in ALS patients compared to healthy controls. These mutations increase the activity of SARM1, significantly reduce NAD^+^ levels, and make neurons less resilient to modest stressors [84]. Expression of a constitutively active SARM1 mutant in neurons was enough to cause motor dysfunction, though whether this will eventually lead to death is still not known [83]. Overall, this suggests that SARM1, while not likely causing ALS to develop, may make individuals more susceptible to ALS and needs to be considered as a therapeutic target in ALS patients.

The exact causal mechanism of ALS has yet to be discovered. However, any treatments for ALS will likely require more than one approach. A combined treatment of NR and pterostilbene had very promising results. SOD1^G93A^ mice had enhanced nerve function, motor behavior, and survival compared to mice receiving only NR or only pterostilbene. This combined treatment also augmented mitochondrial health and the response to reactive oxygen species [92]. What may be even more promising is the response found in human patients. In a pilot study using ALS patients, after 4 months, an NR and pterostilbene treatment significantly slowed the progression of symptoms compared to control patients that received a placebo [109]. Currently, there is an ongoing clinical trial comparing different doses of NR and pterostilbene. The primary aim of this trial is to determine how this treatment impacts ALS progression, with other measures, like quality of life, also being recorded (clinicaltrials.gov: NCT04562831). Until an exact disease mechanism is determined, future investigations should be focused on determining how paired treatments using NAD^+^ precursors may affect ALS progression. Since there are multiple NAD^+^ precursors, determining which one is the best could improve NAD^+^ treatment efficacy.

### 3.2. Charcot–Marie–Tooth Disease

Charcot–Marie–Tooth disease (CMT) is the most common inherited motor and sensory neuropathy, with a worldwide prevalence of 1:2500 people [110]. CMT is a diverse neuropathy with over 80 genes associated with the disease [111]. Symptoms typically present themselves in childhood and early adolescence but may not become apparent until adulthood [110]. CMT can be classified different ways: age of onset, demyelinating or axonal, and affected nerve type (motor, sensory, or both) [110]. CMT has been observed since the 1800s, but despite this, there has not been considerable investigation into the potential of NAD^+^ as a therapeutic remedy, though what has been performed suggests potential benefits.

Crossing CMT rats with WldS rats was beneficial. WldS rats have a chimeric Ube4b-Nmnat1 protein that is mis-localized from the nucleus to the cytoplasm and confers resistance to axon degeneration following injury [112]. CMT-WldS crossed rats had improved axon health and nerve function compared to CMT rats. Though, the direct administration of NAM did not increase axon health [113]. This may indicate that Nampt may be affected given that NAM is a substrate for Nampt. A novel CMT type found in a Chinese family suggested that the mutant protein responsible for CMT developing, DHTKD1, resulted in reduced NAD^+^ and NADH levels and an altered NAD^+^/NADH ratio. However, this group did not investigate the mutant DHTKD1 directly but instead compared the effects of the specific knockdown of DHTKD1 on cellular energetics based on the results that the CMT-affected individuals had significantly lower DHTKD1 levels [114]. Two different CMT models, Mtmr2 and Pmp22, showed improvements following treatment of the NAD^+^ precursor niacin. Niacin ameliorated the myelination defects observed in both Mtmr2 and Pmp22 models [115].

Human fibroblasts carrying a demyelinating CMT type displayed altered mitochondrial bioenergetics and likely had an imbalance in the NADH/NAD^+^ ratio. These fibroblasts also had reduced Sirt1 expression and increasing Sirt1 activity reversed some of the Complex I deficiencies observed in this CMT type [116]. Interestingly, knockdown of Sirt2 in an axonal form of CMT resulted in improved motor performance, increased NMJ number, and extended the lifespan in Drosophila. However, these results may not relate to NAD^+^ availability but instead relate more to the specific mutation that caused CMT. In this specific CMT type, the mutated protein, GARS, binds directly to Sirt2 to affect normal Sirt2 function [117]. The benefits of Sirt2 knockdown in this case would have no relation to NAD^+^ availability.

Overall, much more investigation is needed in order to determine whether NAD^+^ may play a role in CMT progression or in a treatment regimen. More specifically, other NAD^+^ precursor molecules, like NMN or NR, should be investigated as they demonstrate more robust effects than NAM or niacin [118]. Additionally, proteins related to NAD^+^ biosynthesis, especially Nampt, should be investigated more to see whether expression to these important proteins is altered in CMT.

### 3.3. Parkinson’s Disease

Parkinson’s disease (PD) is a progressive neurodegenerative disease characterized by the degeneration of the dopaminergic neurons of the substantia nigra. Although it is not traditionally considered an MND, this degeneration leads to impaired motor control and movement. The motor impairments during PD are generally uncontrolled tremors, slow movements, and muscle stiffness [6,119]. The de novo pathway has been investigated for a role in PD. It has been proposed that targeting the de novo may prove therapeutic, with mutations in ACMSD being linked to PD [120]. Targeting the de novo pathway alone may not be the best approach, however, given that it predominantly occurs in the gut and certain metabolites of the pathway are considered to be neurotoxic. Due to this, focusing on the metabolites or enzymes of the NAD^+^ salvage pathway may be a better therapeutic approach. The de novo pathway may be utilized in a different manner. Metabolites of the de novo pathways, especially kynurenine, may be important in the brain–gut axis, which is the communication between the gut and the CNS [121,122]. As such, measuring the levels of the metabolites of the de novo pathway may be beneficial, where they could potentially be used as biomarkers of disease development and progression [123].

In vitro, iPSCs expressing a PD-associated gene showed reduced NAD^+^ levels, but this was only found after being differentiated into dopaminergic neurons. These dopaminergic neurons also demonstrated impaired ATP-linked respiration [124]. NMN treatment was able to prevent the NAD^+^ and ATP reductions observed in PD-like PC12 cells [125]. Treating neurons with compounds that increased NAD^+^ levels (pyruvate, NAM, NAD^+^, sirtinol) improved neuronal viability in both 6-OHDA and MPTP-treated PD models. This protective effect could be the result of maintaining the NAD^+^/NADH ratio and normal energy metabolism [126]. Increasing NAD^+^ availability may help PD neurons by preventing any detrimental effects due to energy depletion.

Treatments using metabolites of the NAD^+^ salvage pathway may be used for PD treatments. NAM treatment was able to improve mitochondrial function and motor behavior in MPTP-treated and α-synuclein PD models [127]. Parkin and pink1 mutant PD models had reduced levels of NAD^+^, NR, and NMN, but an NAM treatment was able to increase mitochondrial health and prevent degeneration of dopaminergic neurons [128,129]. In vitro and in vivo models for mutant β-glucocerebrosidase (GBA)-induced PD had augmented mitochondrial function, mobility, and NAD^+^ metabolism after NR administration [130]. There may also be a prophylactic role for NAD^+^ in combatting PD. In vitro, NAD^+^ pre-treatment was able to prevent oxidative stress, improve mitochondrial function, and increase cell viability prior to exposure to 6-OHDA. In vivo, an NAD^+^ pre-treatment was able to enhance motor performance and prevent neurodegeneration in mice prior to PD being induced [131]. Other NAD^+^-related metabolites should be examined to determine whether they also could serve in a preventative capacity against the development of PD.

There is direct evidence suggesting Nampt is involved in PD. 6-OHDA-treated PC12 cells had a dose-dependent reduction in Nampt expression and cell survival, with similar reductions to NAD^+^ levels and the NAD^+^/NADH ratio. NMN administration was able to prevent cell death in these PD-like PC12 cells, but Nampt inhibition using FK-866 reduced cell survival even more [55]. Mouse models of PD given P7C3 had improved motor function, increased neurogenesis, and less dopaminergic neurodegeneration [132,133,134]. Unfortunately, Nampt expression levels were not affected in GBA-PD models given NR treatment [130]. Nampt levels could serve as a marker for early or untreated PD in humans [135]. These results indicate that Nampt may be useful in both identifying and treating PD.

Similar to ALS, some of the therapeutic action of NAD^+^ could be achieved by altering the activity of NAD^+^-consuming proteins. Increased Sirt activity appears to have a positive effect against PD. Sirt1, Sirt3, and Sirt6 have all been implicated as both therapeutic targets and biomarkers for PD [136,137,138]. The protective effects from enhanced NAD^+^ availability is possibly due to preventing the reduction in Sirt3 expression [131]. Since Sirt3 is located in the mitochondria, this reduction of Sirt3 may help limit or reverse any mitochondria-associated problems, such as reduced mitochondrial biogenesis, function, and response to oxidative stress [139]. Increased expression of Sirts may not be protective if NAD^+^ levels are also not increased [124]. Parp1 involvement in PD also makes it a likely target for any therapy involving NAD^+^ precursors. Parp1 activity is involved with many of the toxic aspects of PD. Elimination of Parp1 activity reduced degeneration of dopaminergic neurons and improved motor behavior for multiple PD models [140]. A role for Parp1 in PD extends beyond mouse models. In a recent post-mortem study looking at PD brains, Parp1 expression was elevated in the cytoplasm of dopaminergic neurons in the substantia nigra. They also found that this Parp1 mis-localization extended to glia cells [141]. These findings indicate that achieving the greatest benefit from increased NAD^+^ availability may also require the inhibition of Parp activity, specifically Parp1.

The therapeutic potential of NAD^+^ precursors in PD is currently being investigated. In a case study, a 65-year-old PD patient was administered niacin for 45 days followed by an assessment using the Unified Parkinson’s Disease Rating Scale (UPDRS) [142]. The patient exhibited improved motor, cognitive, and sleep measures. He also had significantly reduced GPR109A expression, a marker of neuroinflammation. This same research group then followed with a larger trial with 42 PD patients and examined the effect of daily niacin treatment for 12 months. After the trail finished, the PD patients receiving niacin had improved UPDRS III scores, which is the motor function assessment portion of the UPDRS. Patients also had lower neuroinflammatory markers, GPR109A and NF-κB [143]. There are also clinical trials examining the effect of NR on PD progression. One is examining the effect of NR only on PD progression (clinicaltrials.gov: NCT03816020). This trial appears to be completed though results have yet to be released. A second trial is examining the impact of NR on PD progression when NR is paired with sinemet or selegiline, two drugs approved to treat PD (clinicaltrials.gov: NCT03568968). This trial is still ongoing. These trials should provide insight into how NAD^+^ precursors may be best utilized for treating PD.

### 3.4. Huntington’s Disease

Huntington’s disease (HD) is an inherited autosomal dominant neurodegenerative condition that causes progressive motor deficits, as well as psychiatric and cognitive impairments. HD has a prevalence of nearly 3 per 100,000 people worldwide. HD is caused by the expansion of the cytosine-adenine-guanine (CAG) trinucleotide repeat in the huntingtin gene. The normal number of CAG repeats in the gene is between 6 and 26 and HD typically develops once the number of repeats exceeds 36. The increased number of CAG repeats causes the huntingtin protein to misfold and aggregate inappropriately in the cytoplasm or nucleus. Huntingtin is expressed in many tissues, including highly in the CNS. As the protein aggregates start to accumulate, neuronal function is altered and eventually leads to neuronal death. This neuronal cell death is especially prominent in the striatum. Motor problems may initially begin with fidgeting but eventually gait and posture are also impaired. HD patients also struggle to recreate simple sequences of motor movements, such as in the Luria tri-strip test [144]. Currently, there is no cure for HD and any treatments are aimed at alleviating symptoms [144,145].

In HD, changes in NAD^+^ levels may reflect changes to cellular bioenergetics and may serve as a biomarker for disease progression [146,147,148]. Thus, NAD^+^ metabolism has been investigated as an approach for treating HD symptoms. Treating HD flies with either NAM or niacin was neuroprotective in a dose-dependent manner [149]. B6.HDR6/1 mice treated with NAM had enhanced motor behavior and increased PGC-1α and BDNF mRNA and protein levels [150]. Mice injected with 3-nitroproprionic acid (3-NPA), an inhibitor of complex II of the electron transport chain, develop HD pathological features and have a lower NAD^+^/NADH ratio. Following 3-NPA, NAD^+^ or NAM administration were both able to reduce cell death and improve the NAD^+^/NADH ratio. Mice given NAM had smaller striatal lesions caused by 3-NPA [126]. However, while NAM treatment increased mitochondrial function in YAC128 neurons, long-term administration did not ameliorate any HD behavioral deficits in YAC128 mice [151]. NR and NMN are better tolerated for long-term treatment than NAM so they may be better approaches to assess any long-term therapeutic effects of NAD^+^ augmentation. CAG140 knock-in mice given moderate aerobic exercise had elevated NAD^+^ levels and indications of improved energy metabolism, compared to unexercised CAG140 knock-in mice [152]. There has been little investigation into the impact that NAD^+^ biosynthetic enzymes may have on HD, but the NAD^+^ salvage pathway should be investigated more directly. Overexpression of Nmnat reduced huntingtin aggregation and enhanced mitochondrial and neuronal function. The positive effects of Nmnat overexpression were present whether overexpression was occurring throughout life, or if it was induced at the time of symptom appearance [153]. One lab also reported that, in vivo, NR administration was neuroprotective and activated Sirt1 and Sirt3 [154]. They also reported that NR treatments ameliorated the motor and molecular phenotypes in R6/2 and BACHD mice, two different HD models.

Of the NAD^+^-consuming enzymes, Sirts have been the most widely investigated for treating HD. In an HD Drosophila model, stimulating Sirt1 activity, by administering resveratrol, reduced neurodegeneration, whereas inhibiting Sirt2 was neuroprotective [149]. Administration of the Sirt2 inhibitor, AK-7, enhanced motor performance and decreased huntingtin aggregation in R6/2 and CAG140 knock-in HD mouse models [155]. Interestingly, the knock-out of Sirt2 expression in R6/2 mice did not result in any improvements of behavior or aggregate formation [156]. These conflicting results may be the result of different experimental models.

In N171-82Q mice, resveratrol increased the expression for genes important in energy homeostasis, such as PGC-1α and NRF-1, in the cortex but not the striatum [157]. Resveratrol improved mitochondrial health and motor learning in YAC128 mice [151]. However, resveratrol had no effect on motor behavior or survival [157]. Deletion of Sirt1 from brains of R6/2 mice worsened motor impairment and brain-specific Sirt1 overexpression enhanced striatal volume and motor performance. Sirt1 may also reduce the toxicity of mutant huntingtin in neurons. These improvements from Sirt1 may be occurring due to higher CREB-TORC1 activity, which could in turn upregulate the expression of BDNF [158]. Sirt1 activity is impaired in the striatum of R6/2 mice and is negatively correlated with HD progression [159]. Sirt3 has also been targeted for investigation. Enhanced Sirt3 activity augmented mitochondrial function, possibly by increasing AMPK activity, and elevated the NAD^+^/NADH ratio [160]. There are some inconsistencies between human HD patients and mouse models, however. In human HD patients, Sirt3 expression in the striatum is not changed, while at a late stage of HD in an R6/2 mouse model, Sirt3 expression is significantly increased in the striatum but reduced in the cortex [103]. Altogether, these results indicate that any improvements due to increased NAD^+^ availability would likely involve Sirt1 and/or Sirt3 activity.

Other than Sirts, there are strong indications that Parp activity plays an important role in HD. Like ALS and PD, these results indicate a harmful increase in Parp activity in HD. Parp expression was significantly increased in human HD brains [161]. Inhibiting Parp1 improved the survival of neurons in the striatum of R6/2 HD mice [162]. Parp1 inhibition increased NAD^+^ and ATP availability and reduced markers of inflammation in 3-NPA HD mice. Unfortunately, behavioral impairments were not prevented [163]. These finding indicate that Parp inhibition likely needs to be paired with other treatments to be most effective.

Although the majority of NAD^+^ produced in neurons is generated by the NAD^+^ salvage pathway, there is not much research into how the NAD^+^ salvage pathway may be involved in HD. Given that NR and NMN have had very positive results in treating ALS and PD, these two NAD^+^ precursor molecules should be investigated. Additionally, the expression of Nampt and Nmnat, the two enzymes in the NAD^+^ salvage pathway, should be investigated. If the clinical trials for ALS and PD are any indication, increasing NAD^+^ availability, using NR or NMN, will likely need to be paired with another treatment, such as an Sirt activator, Parp inhibitor, or a previously approved drug for HD treatment, to achieve the most positive results.

### 3.5. Other Motor Neuron Diseases

The role of NAD^+^ or NAD^+^ metabolism in other MNDs has not been well studied, with little to no direct investigation for some diseases, but there are indications that improving NAD^+^ availability may prove beneficial. MNDs can be viewed on a spectrum based on whether upper motor neurons or lower motor neurons are affected. In progressive muscular atrophy (PMA), only lower motor neurons are affected. In primary lateral sclerosis (PLS), only upper motor neurons are affected. ALS is between PMA and PLS, with both upper and lower motor neurons being affected. PLS can often be confused with ALS but is less severe and does not result in death. There has not been much direct investigation into PLS, but recent developments may help to change that. In vitro experiments suggest that energy metabolism and lipid homeostasis are altered in PLS, possibly even more than in ALS [164]. There is a large amount of evidence indicating the benefits of NMN or NR on improving energy and lipid metabolism [9,11]. Research is needed to demonstrate if increased NAD^+^ does serve a therapeutic purpose in PLS. Regarding PMA, there is evidence that PMA may actually be a less aggressive subset of ALS, so the positive results of NAD^+^ augmentation observed in ALS models suggests they would also be effective in PMA conditions, though investigation is needed to demonstrate if this is in fact the case [165].

Spinal and bulbar muscular atrophy (SBMA), also called Kennedy disease, is an X-linked disorder that also primarily affects lower motor neurons. In vitro, SBMA motor neurons exhibit impaired energy homeostasis and significantly reduced ATP levels [166]. Treatments using NAD^+^ precursors can improve cellular energetics and increase ATP levels [9,167]. Spinal muscular atrophy (SMA) affects lower motor neurons and is caused by mutations to the gene survival motor neuron 1. In SMA, there is widespread metabolic dysfunction, impacting glucose, amino acid, and lipid metabolism [168]. In a mouse model of SMA where a drug treatment was paired with nutritional support, SMA mice had increased survival and enhanced skeletal muscle innervation. Among the supplements added to the diet was a vitamin B complex that included niacin [169]. More specific investigation is necessary to determine whether niacin, or other NAD^+^ precursors, may be helpful. However, this should be considered as a positive result. There is also evidence indicating that SMA and ALS, though genetically distinct diseases, are similar in ways that suggest any treatments that are effective against ALS may also be effective against SMA [170].

## 4. Conclusions

A large body of evidence indicates that the reduction of NAD^+^ is a common phenomenon for MNDs. However, changes to NAD^+^ and Nampt might be different in different regions; on the other hand, the regions with high energy consumption, such as the motor cortex, hippocampus, and spinal cord, are particularly susceptible to alterations in NAD availability. In aged brains, lower NAD^+^ levels have been found in the cortex, hippocampus, striatum, cerebellum, and brainstem [39,171,172]. Nampt expression in the brain is also adversely affected with age, with the cortex and hippocampus of aged mice having a significant decrease in Nampt [39]. However, whether there are region-specific NAD^+^ changes for ALS, CMT, PD, HD, or other diseases with motor dysfunction has not been reported. Furthermore, NAD^+^ levels also depend on NAD^+^-consuming enzymes. These need to be investigated further, especially for PD and HD because degeneration occurs in very specific brain regions in these diseases.

The effects of boosting NAD^+^ levels on the outcomes vary greatly depending on the specific disease. There is a considerable amount of research indicating the therapeutic potential of NAD^+^ precursors for treating ALS and PD in animal models. The outcomes of animal studies may be completely different from human trials but will provide insights for clinical trials. In clinical trials on both ALS and PD, NR may be incorporated into other treatments to achieve better outcomes. The outcomes from those trials will hopefully provide a more defined place for NAD^+^ precursors in treating ALS and PD. There is strong evidence indicating that NAD^+^ precursors could be part of a therapeutic approach for treating HD or CMT. HD and CMT are similar in that they are caused by mutations to specific genes, though in the case of CMT, many more genes could be involved. Unfortunately, there has been relatively little direct investigation into how NR or NMN, the two primary metabolites used to increase NAD^+^ levels, may improve HD or CMT symptoms. This research needs to be performed. The evidence for a role of NAD^+^ in less common MNDs is limited. This is due to a lack of direct investigation into these diseases. Given that NAD^+^ precursors are positive in other MNDs, improving NAD^+^ availability in these less investigated MNDs may prove therapeutic as well. In summary, increasing NAD^+^ levels through administration of its precursors could be an effective therapeutic strategy for MNDs.

## Data Availability

Not applicable.
